# Supplementary material for: Assessment of human milk samples obtained pre and post-influenza vaccination reveals a poor boosting of seasonally-relevant, hemagglutinin-specific antibodies
Source: Front Immunol. 2023 May 31;14:1154782. doi: 10.3389/fimmu.2023.1154782 (PMC10264617; doi:10.3389/fimmu.2023.1154782)
Supplement: Supplementary file 1 [file DataSheet_1.docx]

**Supplemental Figure 1: IgA titration curves. (a) 2019-2020 season. (b) 2020-2021 season. Dotted lines indicate pre-vaccine samples, solid lines indicate post-vaccine samples.**

**Supplemental Figure 2: IgG titration curves. (a) 2019-2020 season. (b) 2020-2021 season. Dotted lines indicate pre-vaccine samples, solid lines indicate post-vaccine samples.**

**Supplemental Figure 3: sAb titration curves (anti-SC ELISA). (a) 2019-2020 season. (b) 2020-2021 season. Dotted lines indicate pre-vaccine samples, solid lines indicate post-vaccine samples.**

**Supplemental Figure 4: Seasonally-mismatched titration curves.**
